# Maximum movement and cumulative movement (travel) to inform our understanding of secondary spinal cord injury and its application to collar use in self-extrication

**DOI:** 10.1186/s13049-022-00992-9

**Published:** 2022-01-15

**Authors:** Tim Nutbeam, Rob Fenwick, Barbara May, Willem Stassen, Jason Smith, James Shippen

**Affiliations:** 1 Emergency Department, University Hospitals Plymouth NHS Trust, Plymouth, UK; 2 Devon Air Ambulance Trust, Exeter, UK; 3 Division of Emergency Medicine, University of Cape Town, Cape Town, South Africa; 4 University Hospitals Birmingham, Birmingham, UK; 5 Institute for Future Transport and Cities, University of Coventry, Coventry, UK; 6 Academic Department of Military Emergency Medicine, Royal Centre for Defence Medicine, Birmingham, UK

## Abstract

**Background:**

Motor vehicle collisions remain a common cause of spinal cord injury. Biomechanical studies of spinal movement often lack “real world” context and applicability. Additional data may enhance our understanding of the potential for secondary spinal cord injury. We propose the metric ‘travel’ (total movement) and suggest that our understanding of movement related risk of injury could be improved if travel was routinely reported. We report maximal movement and travel for collar application in vehicle and subsequent self-extrication.

**Methods:**

Biomechanical data on application of cervical collar with the volunteer sat in a vehicle were collected using Inertial Measurement Units on 6 healthy volunteers. Maximal movement and travel are reported. These data and a re-analysis of previously published work is used to demonstrate the utility of travel and maximal movement in the context of self-extrication.

**Results:**

Data from a total of 60 in-vehicle collar applications across three female and three male volunteers was successfully collected for analysis. The mean age across participants was 50.3 years (range 28–68) and the BMI was 27.7 (range 21.5–34.6). The mean maximal anterior–posterior movement associated with collar application was 2.3 mm with a total AP travel of 4.9 mm. Travel (total movement) for in-car application of collar and self-extrication was 9.5 mm compared to 9.4 mm travel for self-extrication without a collar.

**Conclusion:**

We have demonstrated the application of ‘travel’ in the context of self-extrication. Total travel is similar across self-extricating healthy volunteers with and without a collar. We suggest that where possible ‘travel’ is collected and reported in future biomechanical studies in this and related areas of research. It remains appropriate to apply a cervical collar to self-extricating casualties when the clinical target is that of movement minimisation.

## Background

Motor vehicle collisions remain a common cause of spinal cord injury [1]. Following a motor vehicle collision some patients may be able to self-extricate from the damaged vehicle whereas others will need the assistance of the rescue services.

The techniques most frequently utilised by rescue services (e.g. roof removal) have been developed and adopted based upon the principles of movement minimisation and mitigation [2]. This movement focus originates from the understanding that post-injury movements in patients with unstable spinal injuries may exacerbate primary injuries and cause avoidable secondary injury [3]. Whilst some movement is inevitable, the “acceptable” level of spinal movement following an injury is unknown, with prehospital and rescue services often working on the premise that smaller movements are less likely to cause secondary injury than larger movements [4, 5].

Biomechanical studies of spinal movement often lack “real world” context and applicability [6]. Many of the inherent limitations of such studies are both ethically and practically challenging to resolve. Challenges include the unsuitability of data collection technology to operate seamlessly to collect ‘real-world’ data, logistical and ethical concerns associated with using cadaveric models and the inappropriateness of this area of study to the use of an animal model. Despite these challenges, there are practical additional data which can be gleaned from biomechanical studies of healthy volunteers which will be useful to clinicians and those influencing policy in making informed decisions and best judgements in this complex area.

Additional data may enhance our understanding of the potential for secondary spinal cord injury. A variety of biomechanical analysis techniques have been utilised in the study of extrication, episodes of patient care involving the movement of at-risk patients and the effectiveness of immobilisation devices such as cervical collars [4–7]. These biomechanical studies report maximal movement at the cervical spine and utilise this value as a surrogate of the risk of secondary injury. Understanding and reporting maximal movement is appropriate in this context and it is rational that more movement may cause more injury. We suggest that our understanding of the movements during a particular technique could be deepened and therefore a greater appreciation of movement related risk of injury could be improved if all movements during a particular technique or patient movement were understood and reported.

**Fig. 1:**
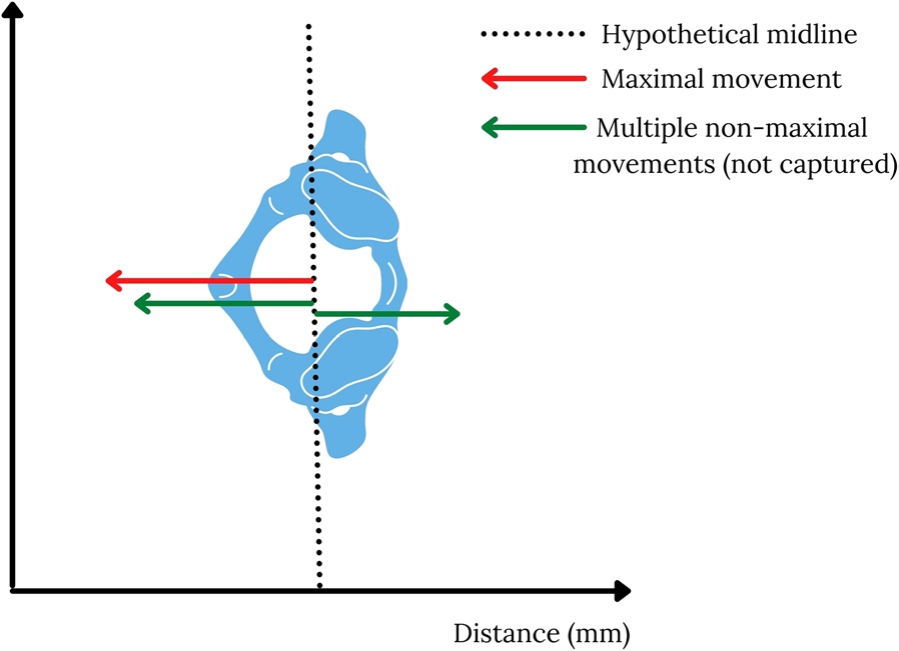
Representation of maximal movements which are captured and reported in current biomechanical models of spinal movement vs non-maximal movements that are not

Secondary spinal cord trauma can be caused by direct damage to the cord itself, (with larger movements expected to cause more damage) and indirect damage to the cord through the initiation or exacerbation of inflammatory processes (which lead to swelling and cord compression) [8]. Similar to many musculoskeletal pathologies a wide range of movements, not just maximal movements, may contribute to the degree of inflammation and/or injury [9]. As such understanding non-maximal movements (particularly repeated movements) will enable a deeper understanding of the effectiveness of spinal movement mitigation and minimisation in relation to its potential to cause secondary cord injury (Fig. 1).

In addition, it is important to understand the movement associated with the application of an immobilisation device or movement minimisation device under study (in this case we use the example of the “in-car” application of a cervical collar). Previous work in this area has considered the value of a collar during extrication [4, 5, 10, 11]. Self-extrication is where a patient exits from the vehicle following a MVC without assistance. Previous data is conflicting on the value of collars during extrication—with most data favouring the application of cervical collars in minimising maximal movement at the cervical spine during self-extrication. Previous studies have not considered the movement associated with application of a cervical collar whilst a patient remains in the vehicle [4, 5, 10, 11].

This study will: (1) Propose the novel metric of “travel” (cumulative movement) (Box 1) (2) Provide data on the movement associated with “in car” application of a cervical collar (3) Use cervical collar application and subsequent self-extrication to demonstrate the utility of ‘travel’.

Box 1: Travel Additional data on movement may be useful when considering the likelihood of a movement or procedure leading to avoidable secondary spinal cord injury. We propose that cumulative movement or “travel” may offer utility in this context.

**Fig. 2:**
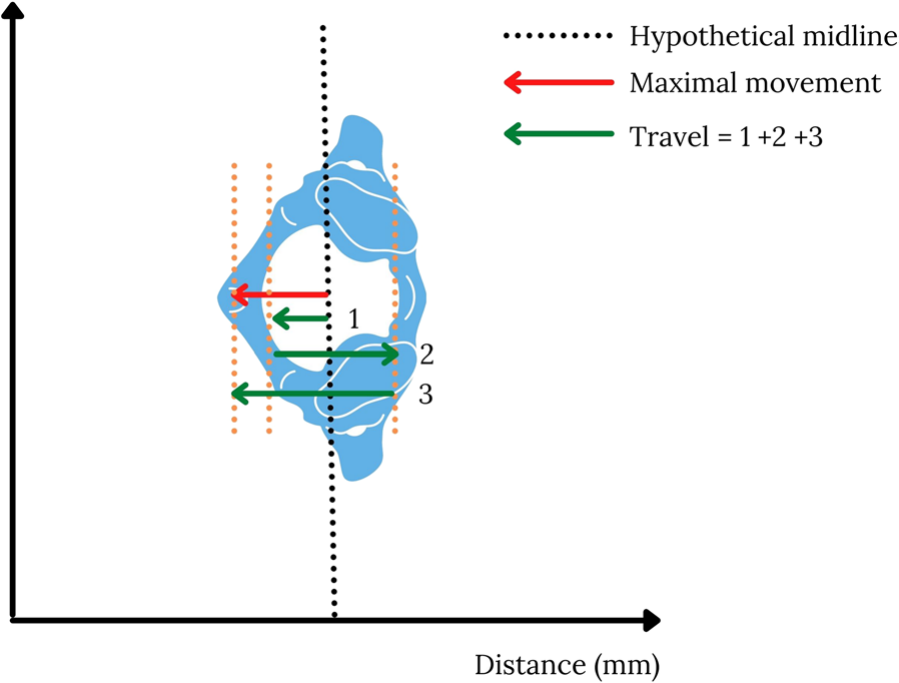
Maximal movement and travel

Travel is the total cumulative movement during the procedure or process and is calculated using the sum of all the incremental movements irrespective of whether the movement is in the positive or negative direction (Fig. 2).

## Methods

We undertook an experimental biomechanical study which considers spinal movement at the cervical spine when a cervical collar is applied “in car” prior to an extrication attempt.

*Participants* Six healthy volunteers were recruited to participate in this study. The volunteers had no previous knowledge of extrication, had no back or neck conditions that may be exacerbated by extrication and had a mass of less than 100 kg. Participants were recruited through local volunteer networks and were not known to the investigating team. Participants were briefed on the study, had access to a participant information sheet in advance and completed written informed consent prior to participation.

*Data collection* Each participant’s height and weight were recorded prior to being fitted with the Inertial Measurement Unit (IMU) (Xsens Awinda; Xsens Technologies B.V., Enschede, Netherlands). The characteristics of IMUs and their suitability for extrication research have been described elsewhere [5]. The IMU sensor was attached to the head using a headband. Sensors were positioned over the clavicular notch on the sternum, and over each scapula using a tight-fitting elastic vest. Orientation data were collected from each sensor via a wi-fi link and sampled at a rate of 40 Hz. The vehicle type was pre-specified as a 5-door hatchback as this represents the commonest vehicle type on UK roads [12]. The vehicle used was a 2010, Peugeot 206.

*Application of collars* The Laerdal (Laerdal Medical Corp., Stavanger, Norway) Stiffneck collars were fitted by one of two members of the study team trained in their use in accordance with manufacturer guidance. Data were collected for 10 applications of the cervical collar for each of the 6 participants (total 60 collar applications). Participants were not wearing any clothing which would hinder collar application and long hair was tied up.

*Analysis* The IMU directly measures the segmental orientations from which relative motions can be calculated and reported, by assuming the relative rotations of adjacent vertebrae across the cervical region are constant. Maximum excursions (movement from a hypothetical midline) were calculated for anterior/posterior (AP) and lateral (Lat) movement of the cervical spine. In addition to reporting maximum excursions (the single largest movement) we report “travel”—the cumulative total of all movements throughout the extrication (Table 1).

Data were captured and analysed using the Biomechanics of Bodies (BoB Biomechanics Ltd, Bromsgrove, UK) software interface before being exported to Excel (Microsoft v. 16.9) and SPSS (IBM v. 25, Armonk NY) for further analysis and reporting [13]. Total excursion, travel, standard deviation and confidence intervals are reported.

We have previously reported data collected using similar techniques which describes maximal movements at the cervical and lumbar spine for self-extrication with and without a collar [5]. A reanalysis of this previously collected data was performed to allow the calculation and reporting of ‘travel’ [5]. Combining the analysis of data collected for both studies allowed for comparative analysis between ‘travel’ for extrication with and without a collar and ‘travel’ associated with collar application.

The study protocol was reviewed and approved by the University of Coventry Research Ethics Committee (reference number P88416).

## Results

Data from total of 60 in-vehicle collar applications across three female and three male volunteers was successfully collected for analysis. A mean (range) age across all of the participants was 0.3 (28–68) years and BMI was 27.7 (21.5–34.6).

**Fig. 3:**
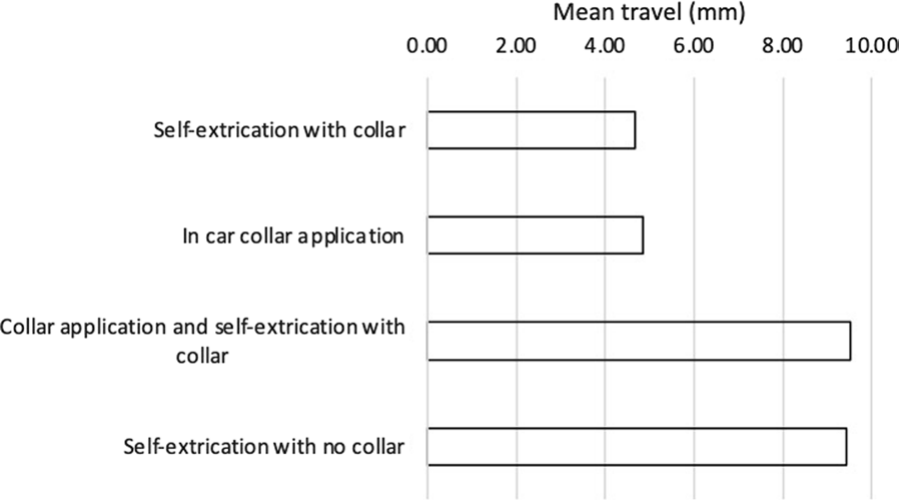
AP travel at cervical spine (mm)

The results are summarised in Tables 1 and 2, and Fig. 3. The mean maximal AP movement associated with collar application was 2.3 mm with a total AP travel of 4.9 mm.

**Table 1:** Participant demographics, mean AP maximal movement and mean AP travel when applying cervical spine collar

Participant	Age	Sex	Height (cm)	BMI (kg/m^2^)	AP movement (maximal) mm^a^	AP movement (travel) mm^a^
1	40	F	167	31.9	3.2	6.7
2	52	F	170	34.6	4.0	9.5
3	57	M	168	31.5	2.2	4.6
4	28	F	167	22.2	2.2	4.5
5	68	M	181	24.4	1.4	2.5
6	57	M	179	21.5	0.7	1.3
Mean	50.3		172	27.7	2.3	4.9

*BMI* body mass index, *AP* anterior posterior movement

^a^Mean movement across ten applications per participant

**Table 2:** Mean travel for self-extrication with and without collar application

	Mean cervical AP travel (mm)	Mean cervical LR travel (mm)	Mean cervical Roll travel (degree)	Mean cervical Pitch travel (degree)	Mean cervical Yaw travel (degree)
Collar application	4.9	2.2	6.5	13.7	20.1
Self-extrication with collar	4.7	4.8	14.0	13.1	12.2
Self-extrication no collar	9.4	6.8	20.1	26.9	26.7
Difference^a^	− 0.1	− 0.1	− 0.5	0.1	− 5.5

^a^Self-extrication with no collar—(Collar application + self-extrication with collar), a negative value indicates larger movement (travel) with collar application and subsequent self-extrication

Figure 3 demonstrates that there is no clinically important difference between cumulative travel across collar application and self-extrication (with collar) when compared to self-extrication without a collar.

## Discussion

We describe a new metric ‘travel’ which we demonstrate provides useful context to biomechanical studies considering movement and the potential risk of secondary spinal cord injury. Across healthy volunteer’s total ‘travel’ is approximately equal across cervical spine movements during extrication with and without a collar. The collar may be most effective for minimising large (maximal) movements though still allowing multiple smaller movements—the cumulative effect of which leads to comparable ‘travel’ [14].

The strength of this new metric is that it allows for the understanding of cumulative movement across an experimental episode (e.g. a single extrication) and as such allows for contextualisation of both total movement and maximal movement when considering potential risk of secondary spinal injury. We suggest that this metric will be particularly useful for comparing extended or complicated biomechanically important procedures (e.g. ‘traditional’ roof-off extrications). When using IMUs collecting data at a moderate frequency (in this case 40 Hz) no additional data capture is required. The weakness of this metric is that like all biomechanical acquired metrics used in this field of research, it lacks direct clinical correlation—though it remains likely that a smaller ‘travel’ will result in a lesser degree or likelihood of spinal injury. This study relies on a small group of uninjured volunteers who have not experienced a recent MVC, who have had obstructions (coats and hair minimised and with collars applied by two experienced clinicians). These conditions are very different from those experienced in a ‘real life’ MVC and as such affect the external validity of the results when applied to casualties following a MVC.

Although we identified that travel was approximately equal across self-extricating volunteers in this study, we have previously demonstrated that maximal movement is larger when a collar is not used (6.9 mm AP with collar, 28.3 mm no collar) [5]. Maximal movement remains an important metric when considering the risk of secondary spinal injury.

## Conclusion

‘Travel’ is a useful metric in understanding total movement in biomechanical research. Total travel is similar across self-extricating healthy volunteers with and without a collar.

We suggest ‘travel’ is collected and reported in future biomechanical studies in this and related areas of research. It remains appropriate to apply a cervical collar to self-extricating casualties when the clinical target is that of movement minimisation.

## Data Availability

The datasets used and/or analysed during the current study are available from the corresponding author on reasonable request.
